# Exploring the role of psychological needs in dating victimization and relationship satisfaction: a mediation analysis among Turkish young couples grounded in self-determination theory

**DOI:** 10.1186/s40359-026-03988-7

**Published:** 2026-01-20

**Authors:** Seda Donat Bacıoğlu, Adem Kantar, Fatmagul Gurbuz-Akcay

**Affiliations:** 1 Department of Educational Sciences, Guidance and Psychological Counseling Program, Trakya University, Edirne, Türkiye; 2 Dr. Adem KANTAR, Department of Psychology, Erzurum Technical University, Erzurum, Türkiye

**Keywords:** Psychological dating violence victimization, Psychological needs, Relationship satisfaction, Self-determination theory

## Abstract

**Background:**

Dating violence is a significant public health problem that adversely affects individuals’ psychological well-being and relationship functioning. Although previous studies have documented the association between psychological dating violence victimization and relationship satisfaction, the psychological mechanisms underlying this association remain insufficiently understood, particularly in non-Western cultural contexts.

**Methods:**

This cross-sectional study was conducted with 401 young adults who had been involved in a romantic relationship within the past six months. Participants completed measures assessing psychological dating violence victimization, basic psychological needs (autonomy, competence, and relatedness) as defined by Self-Determination Theory, and relationship satisfaction. A multiple mediation analysis was performed using bootstrapping procedures.

**Results:**

Psychological dating violence victimization was negatively associated with relationship satisfaction. Among the basic psychological needs, only relatedness significantly mediated this association. Autonomy and competence were not found to be significant mediators. The indirect effect through relatedness was negative, indicating that lower relatedness was associated with lower relationship satisfaction.

**Conclusions:**

The findings suggest that relatedness plays a key associative role in the link between psychological dating violence victimization and relationship satisfaction. Supporting the need for relatedness may help weaken the negative association between victimisation experiences and relationship satisfaction among young couples.

## Introduction

Dating violence is regarded as a phenomenon that is a serious threat to public health in developed societies. This is because the consequences of dating violence can lead to deterioration in victims’ physical (e.g., injuries and diseases such as HIV) [1] and psychological health (conditions such as substance abuse, depression, post-traumatic stress disorder, anxiety, hopelessness, and poorer quality of life) [2–4]. Additionally, factors such as dating violence, making it difficult to establish long-term relationships, causing social isolation, and increasing the risk of being a repeat victim or perpetrator also need to be considered [5].

Dating violence is a broad concept that encompasses various acts of violence within the dating experience, categorized under: physical dating violence (behaviors that violate a partner’s bodily integrity, such as hitting or pushing), psychological dating violence (communication styles based on humiliation, control, disapproval, hostility, neglect, dominance, intimidation, direct threats of violence, and jealousy, related to the attitudes and behaviors of partners), and sexual dating violence (rape, attempted rape, unwanted sexual touching, and other non-contact sexual acts committed without the individual’s consent) [6–9]. Studies indicate that a significant number of adolescents and young people experience violence in dating relationships [10] and they are most frequently exposed to psychological dating violence [11, 12]. Systematic review studies report varying levels of violence: physical violence from 3.8% to 41.9%, physical violence victimization from 0.4% to 57.3%, psychological violence from 4.2% to 97%, psychological violence victimization from 8.5% to 95.5%, sexual violence from 1.2% to 58.8%, and sexual violence victimization from 0.1% to 64.6% [13–15]. Another study indicates that the prevalence of psychological dating violence ranges from 17% to 88% [16]. There is evidence that over 80% of university men and women have been exposed to psychological dating violence [17]. Karatay et al. [18] report that psychological dating violence behaviors, such as use of force, pressure, control, humiliation, and restriction in dating relationships among Turkish university students, are a significant problem. Current studies highlight that psychological dating violence is the most common form of dating violence [19], encouraging researchers to prioritize this area.

In addition to its widespread occurrence, psychological dating violence is mainly bidirectional and exhibits enduring effects [20–23]. Victimization rates are between 76% and 87% for men and 78% to 82% for women, while perpetration rates range from 74% to 85% for men and 83% to 90% for women [24]. These elevated rates in both directions suggest a substantial likelihood that individuals who are victims are concurrently perpetrators. Moreover, psychological abuse within a relationship is predictive of long-term depressive symptoms and anxiety [25]. Compared to other forms of violence, evidence of psychological dating violence are harder to identify [26]. For instance, restricting or controlling a partner’s behavior and withholding emotional support and love can be regarded as psychological dating violence [27]. In his definition, O’Leary [28] also described verbal aggression as including acts of control, domination, humiliation, and repeated criticism of the partner. Marshall [29] introduced a new perspective to psychological dating violence research by distinguishing between overt and subtle forms of abuse. Furthermore, psychological dating violence can occur even in loving, playful, and affectionate situations. Repetitive messages and actions intended to belittle and isolate the partner are another form of psychological dating violence [30]. Historical social roles and different cultural norms can make it challenging to define psychological dating violence [27].

A critical consequence of dating violence is its impact on victims’ satisfaction with their relationships with their abusers [31]. While some studies report a negative relationship between psychological dating violence victimization and relationship satisfaction [32], other studies provide compelling evidence that partners continue to experience relationship satisfaction despite suffering widespread abuse [33]. In this context, it can be seen that this type of violence is a more accepted and common behavior in damaging dating relationships [34]. In other words, psychological dating violence, because of its often unnoticed nature, may not cause dissatisfaction within relationships nor prompt individuals to seek help or consider ending the relationship. In this regard, it can act as a “silent danger,” emphasizing the importance of its study in academic and clinical contexts.

### Relationship satisfaction and basic psychological needs

Relationship satisfaction typically refers to the extent to which individuals feel positive about their relationship and partner [35]. Research on relationship satisfaction generally focuses on married couples [36–38]. However, before marriage, relationship satisfaction is a significant predictor of relationship quality and future time orientation [39]. Young adulthood, in particular, is a critical period during which individuals form their attitudes, habits, and beliefs regarding dating relationships. Healthy dating relationships positively influence young people’s identity development by fulfilling their needs and determining the quality of close relationships formed in adulthood [40].

Factors affecting relationship satisfaction include the perspectives on the relationship, relationship conflicts and their solutions [41], and meeting of partners’ basic psychological needs [42]. In this study, basic psychological needs are discussed from the perspective of the Self-Determination Theory. The reason is that self-determination theory can serve as a basis for developing and enhancing the functionality of romantic relationships characterized by choice and high motivation [43]. This theory is an empirically based psychological theory that attempts to explain human behavior and development within the framework of motivational processes [44]. The theory assumes that human behavior, as a social phenomenon, emerges from interaction with environmental factors [45]. The primary purpose of the theory is to determine under what conditions humans’ innate capacities develop and which conditions hinder their development, taking into account biological, social, and cultural factors [44, 46].

According to self-determination theory, humans have three basic psychological needs that support the pursuit of life goals: personal growth, social integration, and well-being [45]. These are the needs for autonomy, competence, and relatedness. Autonomy emphasizes the reality of behaviors and choices that are compatible with the individual’s needs [47] and support their welfare and relationship maintenance [43, 48]. Individuals with greater relationship autonomy are more supportive of their partners and give more pro-relationship responses to partner transgressions [49]. On the other hand, individuals with low relationship autonomy may interpret their partner’s negative behaviors (e.g., nervous, critical, accusatory, angry) as a personal attack, judgment, or expectation, and may show respond with defense, withdrawal, or counterattack [50]. Competence concerns an individual’s confidence in their own capacity. Satisfying the need for relatedness supports the functioning of the relationship and the sense of belonging and attachment [48]. Meeting the need for relatedness is associated with higher relationship satisfaction [51]. Autonomy and relatedness in close relationships can serve as strategies for maintaining the relationship by promoting compromise when conflicts arise or when partners are confronted with each other’s negative behaviors [31]. In general, high relationship satisfaction is characterized by positive feelings and attitudes towards the partner, and individuals with high relationship satisfaction indicate that they feel their partners meet their needs [52].

### Psychological dating violence victimization and basic psychological needs

The basic psychological needs theory proposed by Deci and Ryan [45] highlights the importance of three fundamental needs for human motivation and well-being: competence, relatedness, and autonomy. Victims of psychological dating violence may experience deficits in all of these basic needs. For instance, dating violence during premarital years can establish an incompatible relationship pattern that continues into adulthood [53, 54]. Psychological dating violence encompasses behaviors such as disrespect, verbal aggression, unwarranted jealousy, humiliation, and exertion of control. This form of violence hampers effective communication and undermines the positive feelings that love provides [30]. For adolescents and young people, dating serves as a form of social connection. The supportive surroundings in dating relationships can help fulfill basic psychological needs, thereby encouraging the pursuit of life goals. However, exposure to frustrating or adverse social conditions may inhibit these psychological needs [55].

The literature includes studies on psychological dating violence, basic psychological needs, and partner dynamics. Psychological dating violence is negatively associated with all aspects of basic psychological needs (competence, relatedness, and autonomy) [56]. In addition to psychological dating violence preventing individuals from fulfilling their basic needs, the fulfillment or lack of fulfillment of these needs can also influence dating violence. A lack of competence, one of the fundamental psychological needs, increases the likelihood of engaging in cyber dating abuse, while satisfying competence reduces it [57]. Petit et al. [58] demonstrated that fulfilling women’s basic psychological needs serves as a protective factor against men’s domestic violence. Another study with female participants confirmed that autonomy directly impacts psychological and physical intimate partner violence, indicating that women in relationships with shared decision-making are likely to experience less intimate partner violence [59]. These findings suggest a negative reciprocal relationship between the two constructs. Previous studies have indicated that the satisfying basic psychological needs mediates the relationship between a negative environment (such as stressful life events or peer victimization) and psychological outcomes [60, 61]. A study conducted by Young-Jones et al. [62] with university students showed that bullying victims experienced significantly lower satisfaction of autonomy and competence needs compared to non-victims. Another study involving 1,845 students in Spain found that being bullied was linked to lower levels of autonomy, competence, and relationship satisfaction [63]. Recent studies, including a study conducted on a Turkish sample, has also demonstrated that autonomy and relatedness needs serve a regulatory function in the relationship between partner abuse and relationship satisfaction [41] Additionally, another study found that frustration with basic psychological needs mediates the negative relationship between perceived partner responsiveness, positively associated with the fulfillment of fundamental needs, and cyberstalking [64].

### Turkish contect

In Turkish culture, it is emphasized that maintaining relationships through coercion, suppression versus control behaviors, humiliation, and restrictions constitute significant issues encountered by university youth aged 18 to 24 [18]. In Toplu-Demirtaş’s [65] study, 85.2% of women and 80.3% of men reported engaging in behaviors aimed at restricting and controlling their partners. Another study found that 67% of men reported being subjected to psychological violence by their partners, compared to 73% of women [66]. These findings show a high prevalence, supporting international research. In her study, Üstünel [67] highlights the need for ongoing research on dating relationships in university settings in Türkiye, raising awareness about different forms of dating violence, challenging attitudes that normalize violence, and implementing informative interventions, especially focusing on psychological violence. Aricioglu and Kaya [41] point out that abusive behavior in romantic relationships, similar to psychological dating violence, is a risk factor for relationship satisfaction. They also emphasize the importance of meeting relationship needs. In this context, the Turkish setting provides an environment to explore the connections between psychological dating violence, basic psychological needs, and relationship satisfaction.

### The present study

Meeting psychological needs is a factor that increases relationship satisfaction. However, there is limited information about how psychological dating violence victimization, which harms relationship satisfaction, affects an individual’s basic psychological needs. It is emphasized that experiencing psychological dating violence at a young age can damage psychological health, hinder the satisfaction of basic psychological needs, and serve as a risk factor for romantic relationships or domestic violence in adulthood. Therefore, promoting healthy partnerships requires examining psychological dating violence victimization. This study aims to explore the role of basic psychological needs in the relationship between psychological dating violence victimization and relationship satisfaction. In this context, the following research hypotheses were tested.

### Hypotheses of the study

H_1_. The need for autonomy mediates the relationship between psychological dating violence victimization and relationship satisfaction.

H_2_. The need for competence mediates the relationship between psychological dating violence victimization and relationship satisfaction.

H_3_. The need for relatedness mediates the relationship between psychological dating violence victimization and relationship satisfaction.

## Method

### Research method

The main aim of this study is to examine the multiple mediating roles of basic psychological needs in the relationship between psychological dating violence victimization and relationship satisfaction. To achieve this primary aim, a correlational research design employed.

### Procedure

The study was conducted in Türkiye during the 2022–2023 academic year. Data were collected from university students across various faculties via an online survey platform. Participants were reached via social media announcements and university student networks. Data collection was carried out remotely, and participation was entirely voluntary and anonymous. To be included in the study, participants were required to be university students and to have had a romantic relationship within the past 6 months. The measurement tools used in the study were obtained after permission was granted by the researchers who developed or adapted them before data collection. Ethical approval for the study was obtained from the ethics committee of a state university in Türkiye. Participants received an informed consent form before participating in the study, which explained the study’s purpose, data confidentiality, and the voluntary nature of their participation.

### Data collection tools

#### Psychological dating violence questionnaire (PDV-Q)

The PDV-Q developed by Ureña et al. [30] and adapted to Turkish by Donat Bacıoglu et al. [68], was used in this study. The purpose of the scale is to measure the levels of psychological violence experienced by individuals in dating relationships. The PDV-Q includes items covering both overt and subtle types of psychological dating violence. Participants are asked to score each item on a 5-point Likert-type scale. Example items are: “To criticize in public or privately” and “Try to control or impede with comments something that the partner wants to do”. There are no reverse-scored items in the scale. High scores obtained from the scale indicate that individuals have high levels of exposure to psychological violence in a dating relationship. Previous studies have provided strong evidence for the construct validity and internal consistency of the Turkish version of the PDV-Q, with Cronbach’s alpha coefficients reported above 0.80 [68].

#### Basic psychological needs scale (BPNS)

The BPNS was developed by Deci and Ryan [45] and adapted into Turkish by Kesici et al. [69]. It is used to determine the individual’s basic psychological needs. This scale consists of three subscales to measure “Autonomy”, “Competence”, and “Relatedness” needs. Example items include: “People I interact with on a daily basis tend to take my feelings into consideration” and “People are generally pretty friendly towards me.” As the scores individuals receive from the subscales increase, their perceived satisfaction with the relevant psychological need increases. Conversely, when scores decrease, the degree of experiencing the relevant psychological need increases. The Turkish version of the scale has demonstrated satisfactory validity and reliability across different samples, with acceptable internal consistency coefficients reported for all three subscales [69].

#### Relationship Assessment Scale (RAS)

The RAS is a scale developed by Hendrick [70] to evaluate general relationship satisfaction, and was adapted into Turkish by Curun [71]. This scale comprises seven items, and participants evaluate each statement on a 5-point Likert-type scale. Example items include: “Compared to others, how good is your relationship?” and “How much do you love your partner?” The analyses conducted in the original study revealed that the scale had a single dimension and had high reliability and criterion-related validity. In short, this shows that it is a brief, psychometrically valid and reliable instrument for measuring relationship satisfaction. An increase in RAS scores indicates greater romantic relationship satisfaction. The Turkish adaptation of the RAS has shown high internal consistency and good criterion-related validity in previous studies [71].

### Participant group

Demographic characteristics of the study participants are presented in Table 1.

**Table 1:** Demographic characteristics of the participants group (n = 401)

Variable	Group		f	%
Gender	Female		244	60.8
	Male		157	39.2
Faculty	Literature		30	7.6
	Education		150	37.2
	Economics and Administrative Sciences		55	13.5
	Health Sciences/Medicine		57	14.3
	Engineering		35	8.9
	Other		74	18.5
Grade Level	1		65	16.2
	2		67	16.7
	3		116	28.9
	4		153	38.2
	***Min***	***Max***	***x̄***	***SD***
Age	18	33	21.38	2.00
Length of Romantic Relationship (Months)	1	85	16.37	18.41

As shown in Table 1, the study sample consisted of individuals continuing their university education. The participants’ ages ranged from 18 to 33 (x̄ = 21.38, SD = 2.00). A total of 401 individuals participated in the study, including 244 women (60.8%) and 157 men (39.2%). When the study participant group was examined by faculty type, the largest proportion of participants was from the faculty of education (37.2%). Moreover, when participants were reviewed by grade level, the highest percentage was fourth-year undergraduate students (38.2%). Participants were expected to have been in a romantic relationship within the past 6 months to be included in the study. Accordingly, the average duration of romantic relationships among participants in the study was 16.37 months, with a standard deviation of 18.41.

### Preparation of study data for analysis

#### A priori sample size calculation

A Monte Carlo power analysis was conducted to assess the statistical power to detect indirect effects in the multiple mediation model. Based on the observed model parameters, the results indicate that the current sample size (*n* = 401) provides sufficient power to detect the indirect effect via relatedness. Accordingly, the sample size was considered adequate for testing the proposed mediation model.

Following the evaluation of sample size adequacy, the dataset was examined to ensure that all statistical assumptions required for the planned analyses were met. Accordingly, the data screening and assumption checks were conducted as described below.

#### Analyses and assumption checks

Since it is necessary to work with a high-quality dataset to obtain high-quality research findings, several assumptions and requirements were met before testing the research hypotheses. First, the reverse-scored items for the variables in the research model were recoded. Secondly, it was assessed whether any missing values were present in the dataset. In the third step, outlier control was performed on the study variables. Accordingly, unidirectional outliers ​​were examined with the standard Z score, histogram, and boxplots, while multidirectional outliers ​​were reviewed with the Mahalanobis distance value. Data for 5 participants identified as outliers were excluded from the analysis. The study variables were assessed for normality using the skewness and kurtosis coefficients.

The SPSS PROCESS Macro plug-in was used to test the research hypotheses. “Model 4”, one of the mediation models proposed by Hayes [72], was selected in the statistical program, and the analyses were carried out accordingly. The mediation analyses proposed by Hayes [72], using the bootstrapping method, are based on least-squares regression analysis. Therefore, before proceeding with the mediation analyses, it was checked whether the dataset was suitable for regression analysis by examining multicollinearity, variance inflation factor (VIF), tolerance, and Durbin-Watson (DW) values. In the examinations, all VIF values were less than 10, and tolerance values were greater than 0.10. Furthermore, a DW value of 1.87 was obtained. When the transferred values ​​were evaluated together, it was seen that the data obtained from the study sample were suitable for performing mediation analyses.

## Findings

Descriptive statistics for research variables are shown in Table 2.

**Table 2:** Descriptive Statistics for Study Variables (n = 406)

Variables	x̄ ± SD	Skewnes	Kurtosi	1	2	3	4	α	ω
Psychological Dating Violence Victimization	19.16 ± 5.27	1.06	.92	-.27*	-.22*	-.10*	-.16*	.80	.81
Relationship Satisfaction (1)	33.55 ± 5.36	-1.32	1.64	-	.17*	.15*	.25*	.87	.88
Autonomy (2)	22.98 ± 3.90	-.33	-.37	-	-	.50*	.52*	.66	.67
Competence (3)	21.11 ± 4.20	-.22	.27	-	-	-	.56*	.67	.68
Relatedness (4)	35.60 ± 5.80	-.42	-.31	-	-	-	-	.76	.79

*p < .05

**Table 3:** Multiple regression analysis predicting relationship satisfaction

Predictor	B	SE	t	*p*
Psychological Dating Violence Victimization	− 0.208	0.043	−4.85	< 0.001
Autonomy	0.022	0.059	0.376	> 0.05
Competence	0.027	0.057	0.472	> 0.05
Relatedness	0.196	0.061	3.21	< 0.05

When Table 2 is examined, it can be seen that the variable of psychological dating violence victimization is significantly negatively correlated with the variables of relationship satisfaction (*r* = −.27, *p* <.05), autonomy (*r* = −.22, *p* <.05), competence (*r* = −.10, *p* <.05) and relatedness (*r* = −.16, *p* <.05). Furthermore, the relationship satisfaction variable was found to be significantly positively correlated with the variables of autonomy (*r* =.17, *p* <.05), competence (*r* =.15, *p* <.05) and relatedness (*r* =.25, *p* <.05). In addition, since the skewness and kurtosis coefficients of the research variables are within the range of + 2 to −2, it can be said that the study variables are normally distributed [73]. Psychological Dating Violence Victimization measured α = 0.80, ω = 0.81; Relationship Satisfaction α = 0.87, ω = 0.88; Autonomy α = 0.66, ω = 0.67; Competence α = 0.67, ω = 0.68; Relatedness α = 0.76, ω = 0.79. These values indicate that the reliability levels of the measurement tools are acceptable in the research sample [74].

**Fig. 1:**
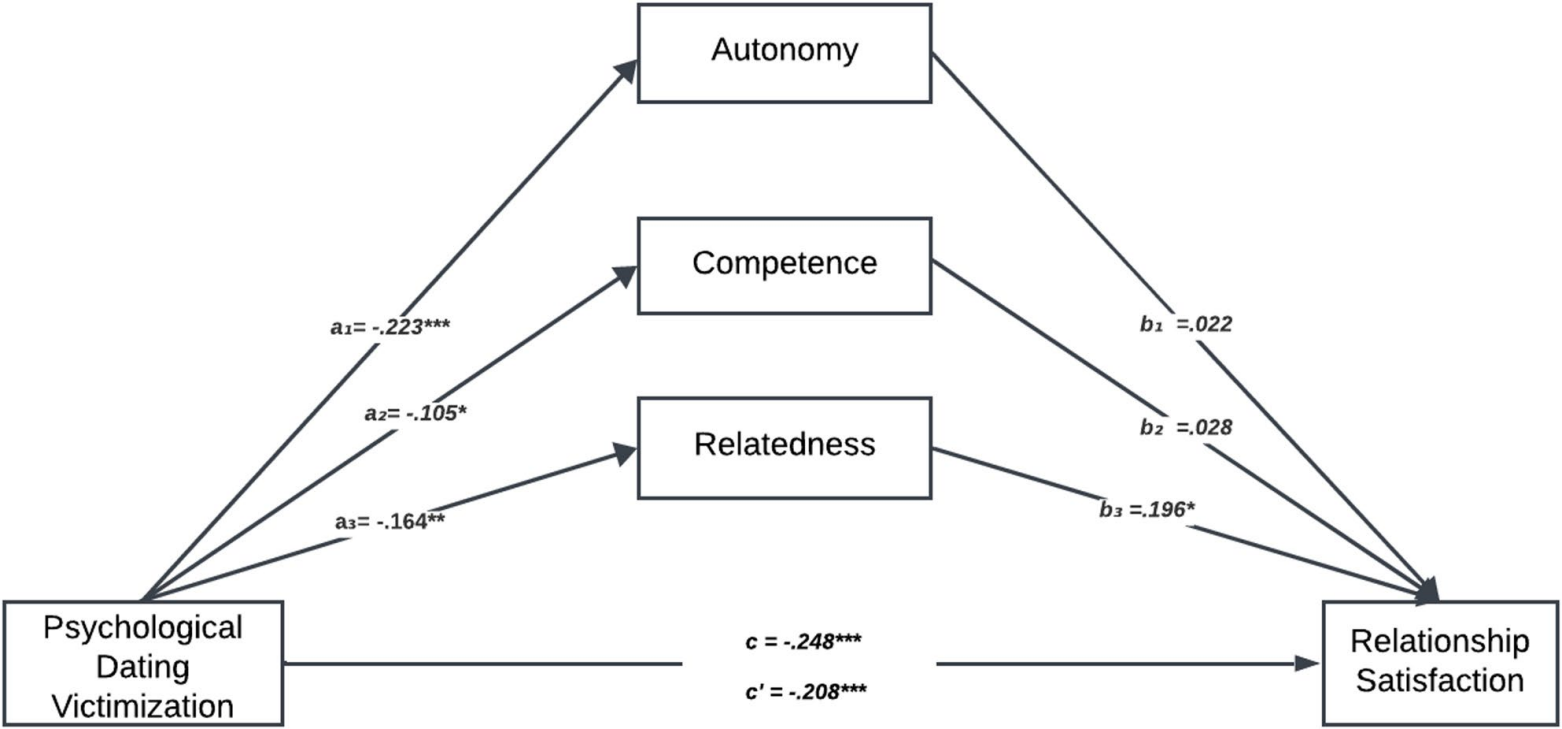
Psychological dating violence victimization and relationship satisfaction: multiple mediating role of basic psychological needs. ***p < .001, **p < .01, *p < .05; c = total effect, c’ = direct effect; standardized coefficients are reported

The multiple mediation model was tested using PROCESS Macro Model 4 with bootstrapping. Indirect effects were evaluated using 5,000 bootstrap resamples and 95% bias-corrected confidence intervals. An indirect effect was considered statistically significant when the confidence interval did not include zero. Unstandardized regression coefficients (B), standard errors, t-values, and confidence intervals were reported for all direct and indirect paths. In addition, the total, direct, and indirect effects were examined to assess the magnitude and significance of the mediation effects (Fig. 1).

When the results of the multiple mediation analysis are examined, the established model is significant (*F*(4;396) = 12.426, *p* <.001) and the independent variables explain 11% of the total variance in relationship satisfaction (*R*^*2*^ = 0.111). Before the mediating variables are included in the model, the total effect of psychological dating violence victimization on relationship satisfaction alone is negatively significant (*B* = − 0.248, *t* = −5.59, *p* <.001) (Table 3). 

After adding the mediating variables to the model, the direct effect of the psychological dating violence victimization variable on relationship satisfaction is again negatively significant (*B* = − 0.208, *t* = −4.85, *p* <.001). When the other paths in the model are examined, psychological dating violence victimization significantly negatively predicts the variables of autonomy (*B* = − 0.223, *t* = −4.57, *p* <.001), competence (*B* = − 0.105, *t* = −2.10, *p* <.05) and relatedness (*B* = − 0.164 *t* = −3.33, *p* <.01). However, the relationship satisfaction variable is positively predicted by relatedness (*B* = 0.196, *t* = 3.21, *p* <.05). On the other hand, the other basic psychological needs, namely autonomy (*B* = 0.022, *t* = 0.376, *p* >.05) and competence (*B* = 0.027, *t* = 0.472, *p* >.05) do not significantly predict relationship satisfaction.

According to the bootstrap method, to claim a variable’s mediating role, the confidence intervals for the indirect effect must not include zero. The indirect effects of psychological dating violence victimization on relationship satisfaction via basic psychological needs are shown in Table 4.

**Table 4:** Indirect effects in multiple mediation analysis

Paths	Coefficients	Standard Error	Confidence Intervals (CI, 95%)	
			Lower Limit	Upper Limit
PDVV → Autonomy **→** RS	− 0.005	0.013	− 0.032	0.022
PDVV → Competence **→** RS	− 0.003	0.006	− 0.017	0.010
PDVV → Relatedness **→** RS	− 0.032	0.013	− 0.061	− 0.008

*PDVV* Psychological Dating Violence Victimization, RS = Relationship Satisfaction

When Table 4 is examined, the need for autonomy does not play a mediating role in the relationship between psychological dating violence victimization and relationship satisfaction. Similarly, the need for competence does not play a mediating role in the relationship between psychological dating violence victimization and relationship satisfaction. On the other hand, the need for relatedness mediates the relationship between psychological dating violence victimization and relationship satisfaction (*B* = − 0.032, *CI* = [−0.061, − 0.008]). In this context, the first and second hypotheses of the study are rejected, whereas the third hypothesis is accepted.

## Discussion

This study was designed to examine the relationship between psychological dating violence victimization and relationship satisfaction, and the mediating role of basic psychological needs in the relationship between these constructs. Evidence of a negative relationship between psychological dating violence victimization and relationship satisfaction has been supported by many studies in the literature [75–79]. Although previous studies often report a correlation between these two variables, it is also important to examine the variables that mediate this relationship.

The analysis carried out within the scope of this study shows that the relationship between psychological dating violence victimization and relationship satisfaction is mediated by the need for “relatedness”, one of the basic psychological needs. This finding reveals that the “relatedness” need of individuals who are victims of psychological dating violence is fulfilled to a lesser extent, which causes their relationship satisfaction to decrease. While this finding does not support the central hypothesis of the Basic Psychological Needs Theory, which posits three basic needs (autonomy, relatedness, and competence), it does support a key hypothesis proposed by the Relationships Motivation Theory [48], a sub-theory of Basic Psychological Needs Theory. Relationships motivation theory contributes to our understanding of the motivational dynamics of people’s close relationships. Relationships motivation theory posits a basic psychological need that drives people to maintain their relationships, namely, the need for relatedness [44, 80]. This need facilitates adaptation to the environment and the satisfaction of basic needs. Therefore, people have had an intrinsic motivation to establish and maintain close and trusting relationships for centuries [42]. As emphasized by the relationships motivation theory, the need for relatedness is one of the most important predictors of relationship quality [51]. It has also been stated that the need for relatedness plays a unique role in ensuring positive relationship functioning [51]. This need entails experiencing a sense of belonging and acceptance within a social context [81]. This need also includes the individual’s urge to establish and maintain close, meaningful bonds with important people in their life [82]. When these statements and the results of the current study are considered together, it can be concluded that individuals exposed to psychological dating violence may not feel close and connected with their partners, and their need for relatedness might not be fulfilled. This can result in low relationship satisfaction. In this context, the direction of the relationships between the variables in this study is generally shown as psychological dating violence victimization → need for relatedness → relationship satisfaction. For instance, in a study using structural equation modeling by Shorey et al. [78], psychological victimization was found to decrease relationship satisfaction. In contrast, Hadden et al. [51] found that fulfilling the need for relatedness predicted higher relationship satisfaction. This study suggests that relationship satisfaction is affected by the need for relatedness and that individuals’ level of satisfaction with the need for relatedness is affected by whether they have been exposed to psychological violence. Healthy fulfillment of individuals’ need for relatedness might interrupt the cycle that leads from psychological dating violence victimization to relationship dissatisfaction.

The other hypotheses of the study aim to examine the mediating role of autonomy and competence needs. The analyses conducted for this purpose showed that the relationship between psychological dating violence victimization and relationship satisfaction was not mediated by the basic psychological needs for “autonomy” and “competence”. Patrick et al. [48] stated that meeting the need for relatedness was the most important predictor of relationship satisfaction, but that the other basic psychological needs, namely the needs for competence and autonomy, also uniquely contributed to it. Guilbault and Philippe [82] also stated that meeting the needs for autonomy, competence, and relatedness predicted psychological well-being in romantic relationships. Contrary to these findings, the data collected in this study showed that individuals’ satisfaction with their needs for autonomy and competence did not significantly predict relationship satisfaction. The findings of the current study are consistent with those of Reis et al. [83]. In their study, Reis et al. [83] examined whether satisfaction of the need for relatedness contributed to emotional well-being independently of the satisfaction with the needs for autonomy and competence. The findings revealed that the need for relatedness predicted positive affect and vitality, independently of the other two needs. These findings are consistent with the findings obtained in the present study. It can be concluded that individuals who are victims of psychological dating violence have low relationship satisfaction because their relatedness needs, rather than their autonomy and competence needs, are not satisfied. In psychological dating violence victimization, the individual is exposed to behaviors such as disrespect, verbal aggression, unnecessary jealousy, humiliation, etc [30]. It is to be expected that individuals who experience these behaviors will not have their need for relatedness met and will subsequently feel lonely, unsupported, and unable to establish a meaningful bond in the relationship. As these negative situations persist, the individual’s satisfaction with their relationship might decrease.

In a culture known for its dominant collectivist values, such as Türkiye, the mediating role of relatedness, a construct emphasizing social and interpersonal connection over competence and autonomy, in the relationship between psychological dating violence and relationship satisfaction aligns with culturally informed beliefs. A study from Türkiye explored the link between abusive behavior in romantic relationships and relationship satisfaction. It showed that relatedness and autonomy played moderating roles, but this effect was stronger for relatedness [41]. Similarly, a study conducted in Iran, a collectivist society, found that competence, autonomy, and relatedness individually predicted intimate partner violence, with relatedness having a greater predictive power than the combined influence of the other two constructs [56]. These findings underline the importance of cultural context in the mediating role of relatedness. The fact that relatedness, which emphasizes strong interpersonal bonds and aligns with collectivist values, mediates the relationship, rather than autonomy, which stresses individual independence, or competence, characterized by a sense of adequacy and effectiveness, may explain this relationship.

In addition to these explanations, it is essential to consider the current level of satisfaction of the participants’ psychological needs at this life stage, namely, emerging adulthood. According to self-determination theory [44], autonomy and competence are basic needs for human well-being and mental health. These needs are very significant for individuals in emerging adulthood. This period encompasses essential life transitions such as identity formation, role acquisition, and future planning, which increase the importance of feelings of autonomy and competence [84]. It can be argued that the autonomy and competence needs of individuals in emerging adulthood are not fully met due to reasons such as the difficulties of the transition period and the state of in-betweenness. As a transitional period from adolescence to adulthood, this period can lead to identity confusion in individuals [85]. During this period, individuals may experience in-betweenness, feeling neither adolescent nor fully adult. Although emerging adults recognize that adolescence has ended, they have not yet assumed adult responsibilities and roles [85]. This situation can lead to uncertainty about the self and the search for identity. During this period, individuals seek to satisfy their need for autonomy by experimenting with independence, and their need for competence by learning from failures and focusing on successes. However, a decrease in the satisfaction of autonomy and competence needs after experiencing dating violence victimization, and the subsequent reducition in perceived relationship satisfaction, is unlikely at this life stage, when individuals might not yet find these needs fulfilling.

In addition to the above, it is also essential to discuss the extent to which parents in Turkish culture support the satisfaction of the autonomy and competence needs. While autonomy is defined as an individual’s ability to initiate and regulate their own actions, competence refers to the individual’s need to be effective in their behaviors and to feel competent [45]. However, fulfilment of these needs can be hindered by the interventionist attitudes of helicopter parents [86]. Helicopter parenting refers to parents who, regardless of the child’s age, constantly intervene and quickly step in to address any problem [87, 88]. These overprotective parents prevent their children in emerging adulthood from achieving their autonomy needs [88]. A study by Schiffrin et al. [89] found that helicopter parenting behaviors were associated with low satisfaction of autonomy and competence needs among college students. Factors such as the increase in violence and the spread of violent acts in society [90] may contribute to the emergence of helicopter parenting in Turkish culture, as parents might instinctively try to protect their children. However, these overprotective behaviors can impede the development of children’s independence and autonomy [90]. Helicopter parents’ efforts to raise their children as independent individuals may paradoxically foster dependency [91]. In Turkish culture, some overprotective parents may display behaviors like these as an accepted societal norm, influenced by the collectivist structure, as mentioned above, and social norms. This can affect how emerging adults’ needs for autonomy and competence are satisfied, regardless of whether they experience psychological dating violence. This effect manifests as a low level of fulfillment for these needs, driven by factors such as helicopter parenting and the unique characteristics of emerging adulthood.

## Conclusion

In conclusion, this study provides evidence that relatedness need satisfaction is statistically associated with relationship satisfaction in the context of psychological dating violence victimization. Specifically, higher levels of psychological victimization were linked to lower satisfaction with the need for relatedness, which in turn was associated with reduced relationship satisfaction. The negative indirect effect observed in the present cross-sectional study indicates that psychological dating violence victimization is associated with lower satisfaction of the need for relatedness, which is, in turn, associated with lower relationship satisfaction. Importantly, this pattern suggests that relatedness may function as a buffering factor in the association between victimization and relationship satisfaction at the correlational level. These findings indicate that relatedness may constitute a key relational mechanism through which psychological victimization is linked to relationship outcomes. In other words, the study reveals that individuals who are aware of their need for relatedness and make an effort to satisfy this need experience relationship satisfaction, even if they are exposed to psychological dating violence. In this context, mental health professionals should raise awareness in individuals who are victims of psychological dating violence, and in cases with serious consequences, refer them to the appropriate support services. These professionals can also enable individuals to join social support groups to meet their need for relatedness or direct them to new, healthy environments aligned on their interests. This approach allows individuals to satisfy the unmet need for relatedness.

The results of this study should be evaluated in light of certain limitations. First, the most critical limitation is the study’s cross-sectional design. Cross-sectional studies do not allow precise determination of cause-effect relationships between variables. While the findings suggest a mediating role for the need for relatedness, they cannot assess changes in these relationships over time. Therefore future longitudinal studies are needed to confirm the mediating role of the need for relatedness. Another limitation is the use of convenience sampling method. This method is limited in its ability to ensure complete representation of the population. Therefore, there are certain limitations regarding the generalizability of the study’s results. Moreover, another limitation is that this study was conducted only in the context of dating relationships in Turkish culture. This limits the generalizability of the findings to other cultures. Therefore, in the future, it is essential to obtain more generalizable results by collecting data from different cultures.

## Data Availability

Data will be available upon reasonable request. Please contact the corresponding author for access.

## References

[1] Teitelman AM, Ratcliffe SJ, Dichter ME, Sullivan CM. Recent and past intimate partner abuse and HIV risk among young women. J Obstetric Gynecologic Neonatal Nursing: Clin Scholarsh Care Women Childbearing Families Newborns. 2008;37(2):219–27. 10.1111/j.1552-6909.2008.00231.x. 10.1111/j.1552-6909.2008.00231.x PMC3677848 18336447

[2] Duerksen KN, Woodin EM. Technological intimate partner violence: exploring technology-related perpetration factors and overlap with in-person intimate partner violence. Comput Hum Behav. 2019;98:223–31. 10.1016/j.chb.2019.05.001.

[3] Alan Dikmen H, Özaydın T, Dereli Yılmaz S. The relationship between dating violence and anxiety/hopelessness among women students in university. Acıbadem Univ Health Sci J. 2018;9(2):170–6. 10.31067/0.2018.9.

[4] Choi EPH, Wong JYH, Fong DYT. Mental health and health-related quality of life of Chinese college students who were the victims of dating violence. Qual Life Res. 2017;26:945–57. 10.1007/s11136-016-1413-4. 27660071 10.1007/s11136-016-1413-4

[5] Park Y, Mulford C, Blachman-Demner D. The acute and chronic impact of adolescent dating violence: A public health perspective. In: Wolfe DA, Temple JR, editors. Adolescent dating violence: Theory, research, and prevention. Elsevier Academic Press; 2018. p. 53–83. 10.1016/B978-0-12-811797-2.00003-7.

[6] Murphy CM, Hoover SA. Measuring emotional abuse in dating relationships as a multifactorial construct. Violence Vict. 1999;14(1):39–53. 10397625

[7] O’Leary KD, Slep AMS. A dyadic longitudinal model of adolescent dating aggression. J Clin Child Adolesc Psychol. 2003;32(3):314–27. 10.1207/S15374424JCCP3203_01. 12881021 10.1207/S15374424JCCP3203_01

[8] World Health Organization. WHO multi-country study on women’s health and domestic violence against women. World Health Organization; 2002. https://iris.who.int/bitstream/handle/10665/43310/9241593512_eng.pdf?sequence=1.

[9] World Health Organization. (2024). *Violence against women.* World Health Organization. https://www.who.int/news-room/fact-sheets/detail/violence-against-women

[10] Shorey RC, Cornelius TL, Bell KM. A critical review of theoretical frameworks for dating violence: comparing the dating and marital fields. Aggress Violent Behav. 2008;13(3):185–94. 10.1016/j.avb.2008.03.003.

[11] Hébert M, Blais M, Lavoie F. Prevalence of teen dating victimization among a representative sample of high school students in Quebec. Int J Clin Health Psychol. 2017;17(3):225–33. 10.1016/j.ijchp.2017.06.001. 29308070 10.1016/j.ijchp.2017.06.001 PMC5756072

[12] Ybarra ML, Espelage DL, Langhinrichsen-Rohling J, Korchmaros JD, Boyd D. Lifetime prevalence rates and overlap of physical, psychological, and sexual dating abuse perpetration and victimization in a national sample of youth. Arch Sex Behav. 2016;45(5):1083–99. 10.1007/s10508-016-0748-9. 27098763 10.1007/s10508-016-0748-9 PMC7202355

[13] Hossain MM, Sultana A, Fan Q, Ma P, Purohit N. Prevalence and determinants of dating violence: an umbrella review of systematic reviews and meta-analyses. Authorea Adv Online Publication. 2020. 10.31235/osf.io/n6hsk.

[14] Rubio-Garay F, López-González MA, Carrasco MÁ, Amor PJ. The prevalence of dating violence: a systematic review. Psychol Pap. 2017;38(2):135–47. 10.23923/pap.psicol2017.2831.

[15] Stonard KE, Bowen E, Lawrence TR, Price SA. The relevance of technology to the nature, prevalence and impact of adolescent dating violence and abuse: a research synthesis. Aggress Violent Behav. 2014;19(4):390–417. 10.1016/j.avb.2014.06.005.

[16] Leen E, Sorbring E, Mawer M, Holdsworth E, Helsing B, Bowen E. Prevalence, dynamic risk factors and the efficacy of primary interventions for adolescent dating violence: an international review. Aggress Violent Behav. 2013;18(1):159–74. 10.1016/j.avb.2012.11.015.

[17] Dardis CM, Edwards KM, Kelley EL, Gidycz CA. Perceptions of dating violence and associated correlates: a study of college young adults. J Interpers Violence. 2017;32(21):3245–71. 10.1177/0886260515597439. 26246117 10.1177/0886260515597439

[18] Karatay M, Karatay G, Gürarslan-Baş N, Baş K. The attitudes and the behaviours of the university students towards dating violence. J Contin Med Educ. 2018;27(1):62–71.

[19] Liles S, Usita P, Irvin VL, Hofstetter CR, Beeston T, Hovell MF. Prevalence and correlates of intimate partner violence among young, middle, and older women of Korean descent in California. J Fam Violence. 2012;27(8):801–11. 10.1007/s10896-012-9471-z. 23645971 10.1007/s10896-012-9471-z PMC3640577

[20] Carney M, Barner JR. Prevalence of partner abuse: rates of emotional abuse and control. Partn Abuse. 2012;3:286–335. 10.1891/1946-6560.3.3.286.

[21] Lawrence E, Yoon J, Langer A, Ro E. Is psychological aggression as detrimental as physical aggression? The independent effects of psychological aggression on depression and anxiety symptoms. Violence Vict. 2009;24(1):20–35. 10.1891/0886-6708.24.1.20. 19297883 10.1891/0886-6708.24.1.20

[22] Mills CP, Hill HM, Johnson JAD. Mediated effects of coping on mental health outcomes of African American women exposed to physical and psychological abuse. Violence Against Women. 2018;24(2):186–206. 10.1177/1077801216686219. 29332534 10.1177/1077801216686219

[23] Taft CT, O’Farrell TJ, Torres SE, Panuzio J, Monson CM, Murphy M, Murphy CM. Examining the correlates of psychological aggression among a community sample of couples. J Fam Psychol. 2006;20(4):581–8. 10.1037/0893-3200.20.4.581. 17176192 10.1037/0893-3200.20.4.581

[24] Cortés Ayala ML, Bringas Molleda C, Rodríguez-Franco L, Flores Galaz M, Ramiro-Sánchez T, Rodríguez Díaz FJ. Unperceived dating violence among Mexican students. Int J Clin Health Psychol. 2014;14(1):39–47.

[25] Lortkipanidze M, Javakhishvili N, Schwartz SJ. Mental health of intimate partner violence victims: depression, anxiety, and life satisfaction. Front Psychol. 2025;16:1531783. 10.3389/fpsyg.2025.1531783. 40861339 10.3389/fpsyg.2025.1531783 PMC12376295

[26] Öngün E, Ünsal G. Intimate relationships and abuse in university. J Acad Res Nurs (JAREN). 2018;4(1):52–8. 10.5222/jaren.2018.052.

[27] Jordan CE, Campbell R, Follingstad D. Violence and women’s mental health: the impact of physical, sexual, and psychological aggression. Ann Rev Clin Psychol. 2010;6:607–28. 10.1146/annurev-clinpsy-090209-151437. 20192793 10.1146/annurev-clinpsy-090209-151437

[28] O’Leary KD. Psychological abuse: a variable deserving critical attention in domestic violence. Violence Vict. 1999;14(1):3–23. 10.1891/0886-6708.14.1.3. 10397623

[29] Marshall LL. Effects of men’s subtle and overt psychological abuse on low-income women. Violence Vict. 1999;14(1):69–88. 10397627

[30] Ureña J, Romera EM, Casas JA, Viejo C, Ortega-Ruiz R. Psichometrics properties of psychological dating violence questionnaire: a study with young couples. Int J Clin Health Psychol. 2015;15(1):52–60. 10.1016/j.ijchp.2014.07.002. 30487821 10.1016/j.ijchp.2014.07.002 PMC6224787

[31] Kaura SA, Lohman BJ. Dating violence victimization, relationship satisfaction, mental health problems, and acceptability of violence: a comparison of men and women. J Family Violence. 2007;22:367–81. 10.1007/s10896-007-9092-0.

[32] Capaldi DM, Crosby L. Observed and reported psychological and physical aggression in young, at-risk couples. Soc Dev. 1997;6(2):184–206. 10.1111/j.1467-9507.1997.tb00101.x.

[33] Williams SL, Frieze IH. Patterns of violent relationships, psychological distress, and marital satisfaction in a national sample of men and women. Sex Roles. 2005;52:771–84. 10.1007/s11199-005-4198-4.

[34] Linder JR, Crick NR, Collins WA. Relational aggression and victimization in young adults’ romantic relationships: associations with perceptions of parent, peer, and romantic relationship quality. Soc Dev. 2002;11(1):69–86. 10.1111/1467-9507.00187.

[35] Rusbult CE, Martz JM, Agnew CR. The investment model scale: measuring commitment level, satisfaction level, quality of alternatives, and investment size. Personal Relationships. 1998;5(4):357–91. 10.1111/j.1475-6811.1998.tb00177.x.

[36] Askari M, Noah SBM, Hassan SAB, Baba MB. Comparison the effects of communication and conflict resolution skills training on marital satisfaction. Int J Psychol Stud. 2012;4(1):182. 10.5539/ijps.v4n1p182.

[37] Curun F, Çapkın M. Predicting jealousy: the influence of attachment styles, self-esteem, personality traits and marital satisfaction. Stud Psychol. 2014;34(1):1–22.

[38] Schmidt CD, Luquet W, Gehlert NC. Evaluating the impact of the getting the love you want couples workshop on relational satisfaction and communication patterns. J Couple Relatsh Ther. 2016;15(1):1–18. 10.1080/15332691.2014.978061.

[39] Eryılmaz A, Doğan T. The mediator role of need satisfaction between subjective well-being and romantic relationships quality. Eurasian J Educ Res. 2013;53:79–96.

[40] Atak H, Taştan N. Romantic relationships and love. Curr Approaches Psychiatry. 2012;4(4):520–46. 10.5455/cap.20120431.

[41] Aricioglu A, Kaya S. Abusive behaviours in relationships, need satisfaction, conflict styles and relationship satisfaction: mediation and moderation roles. BMC Psychol. 2023;11(160). 10.1186/s40359-023-01202-6. 10.1186/s40359-023-01202-6 PMC10190009 37194041

[42] Tasman DR, Eğer Aydoğmuş M. The importance of basic psychological needs in relationship quality: a review on the basis of Self-Determination theory and main relationship categories. Nesne. 2022;10(24):294–315. 10.7816/nesne-10-24-08.

[43] Knee CR, Hadden BW, Porter B, Rodriguez LM. Self-determination theory and romantic relationship processes. Pers Soc Psychol Rev. 2013;17(4):307–24. 10.1177/1088868313498000. 23921674 10.1177/1088868313498000

[44] Ryan RM, Deci EL. Self-determination theory: Basic psychological needs in motivation, development, and wellness. Guilford; 2017.

[45] Deci EL, Ryan RM. The what and why of goal pursuits: human needs and the self determination of behavior. Psychol Inq. 2000;11(4):227–68. 10.1207/S15327965PLI1104_01.

[46] Ryan WS, Ryan RM. Toward a social psychology of authenticity: exploring within-person variation in autonomy, congruence, and genuineness using self-determination theory. Rev Gen Psychol. 2019;23(1):99–112. 10.1037/gpr0000162.

[47] Weinstein N, Rodriguez LM, Knee CR, Kumashiro M. Self-determined self-other overlap: interacting effects on partners’ perceptions of support and well-being in close relationships. J Res Pers. 2016;65:130–9. 10.1016/j.jrp.2016.10.011.

[48] Patrick H, Knee CR, Canevello A, Lonsbary C. The role of need fulfillment in relationship functioning and well-being: a self-determination theory perspective. J Pers Soc Psychol. 2007;92:434–57. 10.1037/0022-3514.92.3.434. 17352602 10.1037/0022-3514.92.3.434

[49] Hadden BW, Rodriguez LM, Knee CR, Porter B. Relationship autonomy and support provision in romantic relationships. Motiv Emot. 2015;39(3):359–73. 10.1007/s11031-014-9455-9.

[50] Schnarch D. Sex, intimacy, and the internet. J Sex Educ Ther. 1997;22(1):15–20. 10.1080/01614576.1997.11074166.

[51] Hadden BW, Smith CV, Knee CR. The way i make you feel: how relatedness and compassionate goals promote partner’s relationship satisfaction. J Posit Psychol. 2013;9(2):155–62. 10.1080/17439760.2013.858272.

[52] Fincham FD, Rogge R. Understanding relationship quality: theoretical challenges and new tools for assessment. J Fam Theory Rev. 2010;2(4):227–42. 10.1111/j.1756-2589.2010.00059.x.

[53] Exner-Cortens D, Eckenrode J, Rothman E. Longitudinal associations between teen dating violence victimization and adverse health outcomes. Pediatrics. 2013;131(1):71–8. 23230075 10.1542/peds.2012-1029 PMC3529947

[54] Murphy CM, O’Leary KD. Psychological aggression predicts physical aggression in early marriage. J Consult Clin Psychol. 1989;57(5):579–82. 2794178 10.1037//0022-006x.57.5.579

[55] Harbin SM, Kelley ML, Piscitello J, Walker SJ. Multidimensional bullying victimization scale: development and validation. J Sch Violence. 2019;18(1):146–61. 10.1080/15388220.2017.1423491.

[56] Nikrouy F, Mohammadi K, Samavi SA. Structural relationship model of basic psychological needs with intimate partner violence: the mediating role of gender discrimination and self-esteem. J Interpers Violence. 2025;40(5–6):1387–411. 10.1177/08862605241259415. 39066554 10.1177/08862605241259415

[57] Valachová M, Lisá E. Frustrated cyber-abuser: narcissistic traits in the context of the basic psychological needs and cyber dating abuse. Comput Human Behav. 2025;162:108465. 10.1016/j.chb.2024.108465.

[58] Petit WE, Knee CR, Hadden BW, Rodriguez LM. Self-determination theory and intimate partner violence: an APIM model of need fulfillment and IPV. Motiv Sci. 2017;3(2):119–32. 10.1037/mot0000054.

[59] Mpondo F, Ruiter RAC, van den Borne B, Reddy PS. Intimate partner violence and its association with self-determination needs and gender-power constructs among rural South African women. J Interpers Violence. 2016;34(14):2975–95. 10.1177/0886260516664316. 27543301 10.1177/0886260516664316

[60] Ren Q, Jiang S. Acculturation stress, satisfaction, and frustration of basic psychological needs and mental health of Chinese migrant children: perspective from Basic Psychological Needs Theory. Int J Environ Res Public Health. 2021;18(9):4751. 10.3390/ijerph18094751. 33946882 10.3390/ijerph18094751 PMC8124301

[61] Jiang S, Ngai SSY. Social exclusion and multi-domain well-being in Chinese migrant children: exploring the psychosocial mechanisms of need satisfaction and need frustration. Child Youth Serv Rev. 2020;116:105182. 10.1016/j.childyouth.2020.105182.

[62] Young-Jones A, Fursa S, Byrket JS, Sly JS. Bullying affects more than feelings: The long-term implications of victimization on academic motivation in higher education. Soc Psychol Educ. 2015;18:185–200. 10.1007/s11218-014-9287-1.

[63] Santurio JI M, Fernández-Río J, Estrada JA C, González-Víllora S. Connections between bullying victimization and satisfaction/frustration of adolescents' basic psychological needs. Rev Psicodidáct. 2020;25(2):119–126. 10.1016/j.psicod.2019.11.002.

[64] Sönmez İ, Solmazer G. Perceived partner responsiveness and intimate partner cyberstalking: The mediating role of basic psychological need satisfaction and frustration. Nesne. 2022:10(26);540–554. 10.7816/nesne-10-26-01.

[65] Toplu-Demirtaş, E. Psychological aggression perpetration among dating college students: The interplay of societal, parental, and personal factors [Unpublished doctoral dissertation]. Middle East Technical University. 2015.

[66] Toplu Demirtaş E, Hatipoğlu Sümer Z. Psychological, physical, and sexual dating violence in emerging adulthood: Assessment and prevalence. Humanistic Perspective. 2022:4(2);408–432. 10.47793/hp.1079451.

[67] Üstünel AÖ. Equality, safety, autonomy in relationships: Testing the effectiveness of a dating violence prevention program for college students. Turkish Psychol Artic. 2020:23(45);39–41. 10.31828/tpy1301996120191008m000016.

[68] Donat Bacıoğlu S, Gürbüz Akçay F, Kantar A. The validity and reliability study of the Turkish version of psychological dating violence questionnaire. Buca Fac Educ J. 2024:(62);2947–2964. 10.53444/deubefd.1472613.

[69] Kesici Ş, Üre Ö, Bozgeyikli H, Sünbül AM. Temel Psikolojik İhtiyaçlar Ölçeğinin geçerlik ve güvenirliği [Validity and reliability of the Basic Psychological Needs Scale]. VII. Malatya: Ulusal PDR Kongresi Bildiri Özetleri Kitabı; 2003.

[70] Hendrick SS. A generic measure of relationship satisfaction. J Marriage Fam. 1988;50(1):93–98. 10.2307/352430.

[71] Curun F. The effects of sexism and sex role orientation on romantic relationship satisfaction (Unpublished master’s thesis). Ankara: Middle East Technical University; 2001.

[72] Hayes AF. Introduction to Mediation, Moderation, and Conditional Process Analysis: A Regression-Based Approach (Methodology in the Social Sciences) (2nd ed.). New York, NY: The Guilford Press; 2018.

[73] George D, Mallery P. SPSS for Windows Step by Step: A Simple Guide and Reference 17.0 Update. 10th Edition, Pearson; 2010.

[74] Fraenkel RJ, Wallen EN. How to design and evaluate research in education. McGraw-Hill; 2006.

[75] Fournier B, Brassard A, Shaver PR. Adult attachment and male aggression in couple relationships: The demand-withdraw communication pattern and relationship satisfaction as mediators. J Interpers Violence. 2011:26(10);1982–2003. 10.1177/0886260510372930. 10.1177/0886260510372930 20587474

[76] Godbout N, Dutton DG, Lussier Y, Sabourin S. Early exposure to violence, domestic violence, attachment representations, and marital adjustment. Pers Relatsh. 2009:16;365–384. 10.1111/j.1475-6811.2009.01228.x.

[77] Katz J, Kuffel SW, Coblentz A. Are there gender differences in sustaining dating violence?: An examination of frequency, severity, and relationship satisfaction. J Fam Violence. 2002:17(3);247–271. 10.1023/A:1016005312091.

[78] Shorey RC, Febres J, Brasfield H, Stuart GL. Male dating violence victimization and adjustment: The moderating role of coping. Am J Men’s Health. 2012:6(3);218-228. 10.1177/1557988311429194. 10.1177/1557988311429194 PMC3382081 22495552

[79] Testa M, Leonard KE. The impact of marital aggression on women's psychological and marital functioning in a Newlywed sample. J Fam Violence. 2001:16(2);115–130. 10.1023/A:1011154818394.

[80] Deci EL, Ryan RM. Autonomy and need satisfaction in close relationships: Relationships motivation theory. In: Weinstein, N. (eds) Human motivation and ınterpersonal relationships. Springer, Dordrecht; 2014. 10.1007/978-94-017-8542-6_3.

[81] Kowal JP, Fortier MS. Motivational determinants of flow: Contributions from Self-Determination Theory. J Soc Psychol. 1999:139;355–368. 10.1080/00224549909598391.

[82] Guilbault V, Philippe FL. Commitment in romantic relationships as a function of partners’ encoding of important couple-related memories. Memory. 2017:25(5);595–606. 10.1080/09658211.2016.1197943. 10.1080/09658211.2016.1197943 27310766

[83] Reis HT, Sheldon KM, Gable SL, Roscoe J, Ryan RM. Daily well-being: The role of autonomy, competence, and relatedness. Personal Soc Psychol Bull . 2000:26(4);419–435. 10.1177/0146167200266002.

[84] Arnett JJ. Emerging adulthood. A theory of development from the late teens through the twenties. Am Psychol. 2000:55(5);469–480. 10.1037/0003-066x.55.5.469. 10842426

[85] Arnett JJ. Conceptions of the transition to adulthood: Perspectives from adolescence through midlife. J Adult Dev. 2001:8(2);133–143. 10.1023/A:1026450103225.

[86] Filiz A, Doğan A. Helicopter parenting in emerging adulthood: An examination within Self-Determination Theory abstract. Nesne. 2023:11(29);419–436. 10.7816/nesne-11-29-05.

[87] Cline F, Fay J. Parenting with love & logic. The Navigators. 1990.

[88] Kouros CD, Pruitt MM, Ekas NV, Kiriaki R, Sunderland M. Helicopter parenting, autonomy support, and college students’ mental health and well-being: The moderating role of sex and ethnicity. J Child Fam Stud. 2017:26(3);939–949. 10.1007/s10826-016-0614-3. 10.1007/s10826-016-0614-3 PMC6907082 31832009

[89] Schiffrin HH, Liss M, Miles-McLean H, Geary KA, Erchull MJ, Tashner T. Helping or hovering? The effects of helicopter parenting on college students’ well-being. J Child Fam Stud. 2014:23(3);548–557. 10.1007/s10826-013-9716-3.

[90] Somers P, Settle J. The helicopter parent (Part 2): International arrivals and departures. College and University; 2010:86(2);2–9.

[91] Van Ingen DJ, Freiheit SR, Steinfeldt JA, Moore LL, Wimer DJ, Knutt AD, et al. Helicopter parenting: The effect of an overbearing caregiving style on peer attachment and self-efficacy. J Coll Couns. 2015:18(1);7–20. 10.1002/j.2161-1882.2015.00065.x.

